# Acute Rheumatism Confined to the Temporo-Maxillary Joint

**Published:** 1892-01

**Authors:** 


### ACUTE RHEUMATISM CONFINED TO THE TEMPORO-MAXILLARY JOINT.

The British Medical Journal of Dental Science reproduces a report by Dr. Gallipe, published in Le Progres Dentaire, of a case of acute rheumatism confined to the temporo-maxillary joint. It occurred in a patient of rheumatic diathesis. The symptoms were swelling of the cheek on the right side; with redness and pain on pressure; mastication and talking were both painful, with exacerbations at times. Pressure over the temporo maxillary joint caused sharp pain. The upper and lower wisdom-teeth on the same side felt as if raised in their sockets. There had been no tooth-ache. Careful examination of these teeth revealed no decay. The diagnosis was made of an attack of acute rheumatism in the right temporo-maxillary joint, and the patient was put upon salicylate of soda and quinine, rapid recovery resulting. Dr. Gallipe considers that the feeling of raising in the teeth was due to an attack of rheumatism in the alveolodental membrane itself, or rheumatic periodentitis, rather than simply the extension of pain from the affected joint.—*The Lancet—Nashville Jour. Med. and Surg.*

### PAPOID IN DIPHTHERIA.

R Papoid, gr. x.
Aquæ, oz. ss.
M. F. solution.

Kohts and Asch painted diphtheretic membranes with this solution every fifteen or twenty minutes with a soft brush. They found that the oftener the application was made the more rapidly membranes disappeared. Koets treated several hundred cases by this method with the greatest success.

R Papoid, dr. ij.
Beta-naphthol, gr. iij.
Acid hydrochl. dil., gtt. xv.
Aq. destil. ad., ox. iv.

M. ft. solution. Sig. Use carefully and thoroughly by means of hand anatomizer every half hour on throat and through nostrils on posterior nares and pharynx, if deposit extends to these localities. Papoid solutions should be made fresh daily.—Ex.

